# Negative Pressure Wound Therapy—A Vacuum-Mediated Positive Pressure Wound Therapy and a Closer Look at the Role of the Laser Doppler

**DOI:** 10.3390/jcm13082351

**Published:** 2024-04-18

**Authors:** Christian D. Taeger, Clemens Muehle, Philipp Kruppa, Lukas Prantl, Niklas Biermann

**Affiliations:** 1 Department of Plastic, Hand and Reconstructive Surgery, University Hospital Regensburg, 93053 Regensburg, Germany; clemens-muehle@web.de (C.M.); lukas.prantl@klinik.uni-regensburg.de (L.P.); niklas.biermann@klinik.uni-regensburg.de (N.B.); 2 Department of Plastic, Hand and Reconstructive Surgery, Ernst von Bergmann Klinikum, 14467 Potsdam, Germany; kruppaph@gmail.com

**Keywords:** negative pressure wound therapy, positive pressure, laser doppler, perfusion, compression, NPWT

## Abstract

**Background**: Negative pressure wound therapy (NPWT) is an intensely investigated topic, but its mechanism of action accounts for one of the least understood ones in the area of wound healing. Apart from a misleading nomenclature, by far the most used diagnostic tool to investigate NPWT, the laser Doppler, also has its weaknesses regarding the detection of changes in blood flow and velocity. The aim of the present study is to explain laser Doppler readings within the context of NPWT influence. **Methods**: The cutaneous microcirculation beneath an NPWT system of 10 healthy volunteers was assessed using two different laser Dopplers (O2C/Rad-97^®^). This was combined with an in vitro experiment simulating the compressing and displacing forces of NPWT on the arterial and venous system. **Results**: Using the O2C, a baseline value of 194 and 70 arbitrary units was measured for the flow and relative hemoglobin, respectively. There was an increase in flow to 230 arbitrary units (*p* = 0.09) when the NPWT device was switched on. No change was seen in the relative hemoglobin (*p* = 0.77). With the Rad-97^®^, a baseline of 92.91% and 0.17% was measured for the saturation and perfusion index, respectively. No significant change in saturation was noted during the NPWT treatment phase, but the perfusion index increased to 0.32% (*p* = 0.04). Applying NPWT compared to the arteriovenous-vessel model resulted in a 28 mm and 10 mm increase in the venous and arterial water column, respectively. **Conclusions**: We suspect the vacuum-mediated positive pressure of the NPWT results in a differential displacement of the venous and arterial blood column, with stronger displacement of the venous side. This ratio may explain the increased perfusion index of the laser Doppler. Our in vitro setup supports this finding as compressive forces on the bottom of two water columns within a manometer with different resistances results in unequal displacement.

## 1. Background

Negative pressure wound therapy (NPWT) is a highly investigated topic throughout many surgical sciences, but its mechanisms of action are among the least understood [1,2,3,4,5,6,7,8,9]. This leads to several disadvantages, especially when dealing with the optimization of technical or individualized patient settings.

This may be further aggravated if the generally accepted and attributed mechanism of action differs from the process actually taking place [10]. One of the possible reasons for this problem regarding NPWT might be due to its name. NPWT suggests that negative pressure is responsible for the mechanism of action and thus its therapeutic success. However, its success is undisputedly the result of numerous different effects, including interstitial edema reduction, but above all improved blood circulation [11,12,13,14,15,16]. Over the last decade, this has been cited many times, with the initial investigation being performed by Morykwas et al., which postulated an opening of the capillaries resulting in increased perfusion [17].

Numerous confirmatory studies using laser Doppler measurements have been published in the literature, but the overall basic physical effects of a vacuum and spring are hardly mentioned [18,19,20]. Sealing a vacuum over a wound with a flexible foil, unlike cupping therapy, and inserting a sponge as an interface will inevitably lead to local positive pressure. The physical mechanism can thus be explained by the foam’s resilient force in combination with the expansion force properties of the foil and wound.

This phenomenon has been the center of investigation in our previous study and we were able to demonstrate the pressure distribution in a three-dimensional model. Furthermore, we discovered a flow reduction in a simple vessel model when a conventional NPWT system was applied as above [1].

The current literature has concluded that NPWT basically induces ischemia, similarly to how pressure on the skin causes decubital ulcers [21]. This consideration was followed by Kairinos, among others, who dealt intensively with this issue [22,23,24,25]. In his opinion, the increased tissue perfusion detected by the laser Doppler is not a net increase in perfusion, and therefore results need to be interpreted with care [26].

In the present work, the perfusion characteristics of healthy volunteers were traced using the latest generation of laser Doppler probes, including the measurement of a perfusion index. This allows us to differentiate between pulsatile arterial flow and non-pulsatile venous flow. In addition, an in vitro model was constructed to show the different displacement of blood columns in the arterial and venous capillary bed mediated by NPWT. The hypothesis is that a greater displacement and acceleration of venous blood is accountable for the laser Doppler readings with respect to NPWT.

## 2. Methods

This prospective trial was conducted based on ethical approval and in accordance with the 2010 CONSORT (Consolidated Standards of Reporting Trials) guideline and the Declaration of Helsinki. It was registered in a public German trial registry (DRKS, DRKS 000 23516) and approved by the institutional ethics committee (17-852-101).

## 3. Participants

Six female and four male participants (mean age: 26 years [SD 1.3]) were included in our investigation. Previous surgeries on the examination side, diabetes, vascular pathologies, heart disease or hypertension in the preliminary examination were reasons for exclusion. The mean body mass index (BMI) of all patients was 23 (SD 1.1), and blood pressure and heart rate remained constant with a mean of 123/68 mmHg (SD 4.2 and SD 6.1, respectively) and 62 beats/min (SD 6.5). All participants provided written informed consent for participation in this trial.

## 4. Study Protocol

### 4.1. In Vivo Perfusion Measurement

The study protocol included a prospective NPWT (Info V.A.C. TM, KCI Medizinprodukte GmbH, Wiesbaden, Germany) application to the intact skin of the upper thigh. The treatments were performed with the patient in a supine position, with slightly flexed knee joints and with both feet placed on stabilizing blankets, with a cherry pit cushion around the ankles to avoid any movements.

To identify microcirculatory changes, two-millimeter plain laser Doppler probes (O2C, LEA Medizintechnik, Gießen, Germany) with a penetration depth of approximately three millimeters were placed beneath the NPWT dressing on one of the participant’s legs.

The middle of the anterior thigh was chosen for placing the NPWT sponge and the probes were applied in the proximal and distal third. To standardize the sponge position, a line between the anterior superior iliac spine and patella was drawn and the exact middle chosen as the center.

The other leg served as a control group, as the same area on the anterior thigh was chosen for probe placement. A ruler aided the correct placement. If changes in circulation were noted due to patient movements, the measurement was repeated.

Prior to a five-minute NPWT application, a five-minute resting phase was used to adjust blood pressure and heart rate. During the NPWT application, participants were encouraged to relax and limit physical stimuli. In order to detect the microcirculatory restitution, a final five-minute observation phase was added to the experiment.

Regarding O2C measurements, values in arbitrary units (AUs) included capillary blood flow and the relative venous hemoglobin level (rHb). According to the manufacturer’s guidelines, blood flow was measured by multiplying flow and volume, with the rHB content serving as an equivalent to the venous filling level. The plotting of the values through the course of each phase is shown in Figure 2.

The Masimo Rad-97^®^ (Masimo Europe Ltd., Puchheim, Germany) was used for its ability to distinguish between the arterial and venous pulse character, using the perfusion index and oxygen saturation. Figure 3 shows the course of the percentual values over time of the three investigated phases. Again, one probe was placed beneath the NPWT dressing, and one on the contralateral leg serving as a control group. The probe placement was similar to the one mentioned for the O2C.

Accommodation settings included closed windows with a thermostat set to 23 °C (73.4°F), and prevenient caffeine or nicotine intake was prohibited.

### 4.2. In Vitro Vessel Perfusion Model

To simulate the nourishing vessel supply of a soft tissue wound, two rubber tubes measuring four millimeters in diameter and 0.1 mm in wall thickness were embedded in a block of 300 bloom ballistic gel (Type A, TMP-products, Bad Camberg, Germany). The NPWT system was assembled to cover a ten-by-ten-centimeter wound area, leaving approximately one-centimeter tissue in depth between the vessels and the NPWT foam. It was set to continuous suction at 125 mmHg on medium intensity.

**Figure 1:**
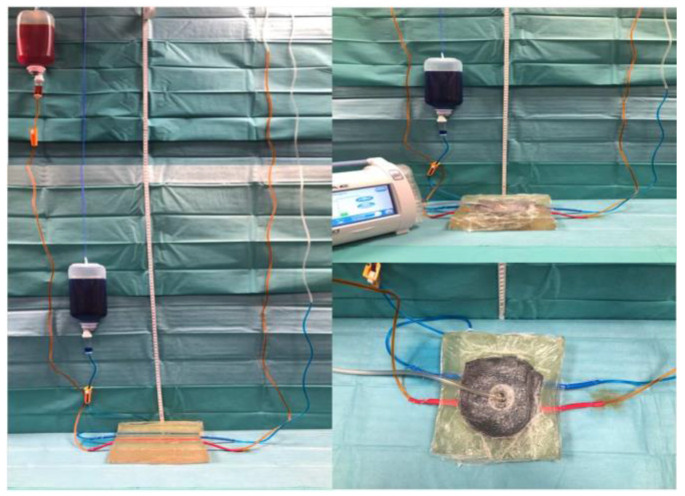
Overview of in vitro arterial and venous vessel model used to show displacing forces of the NPWT system.

The afferent tubes were connected to a standard infusion system (Intrafix^®^, Braun, Germany) and hung at different heights based on a pressure relationship of arterial and venous capillary pressure equal to approximately 1:3. The absolute pressure values of our setup do not mimic the in vivo situation but respect the relative relation of arterial-to-venous perfusion pressure. Food coloring was used to indicate the arterial and venous system (Figure 1).

### 4.3. Statistics

The O2C laser Doppler recorded its measurements in arbitrary units whereas the Rad-97^®^ wrote percentual changes. The measuring interval included a total of three time periods, each consisting of a five-minute frame. The first interval was the participants’ resting phase, which set the baseline for further comparison. This was followed by the NPWT-On and finally the NPWT-Off phase. The Kolmogorov–Smirnov and Shapiro–Wilk tests were used to test variables for normality. A paired *t*-test was then used to analyze the differences between the baseline and the individual NPWT-On and NPWT-Off phases.

A *p*-value < 0.05 was set to indicate significance for all tests. Statistical analysis was carried out using SPSS 25 for Windows (IBM Corp., Armonk, NY, USA).

## 5. Results

### 5.1. Perfusion Measurement with the O2C

Using the O2C beneath the NPWT dressing, a baseline value of 194 and 70 arbitrary units was measured for the flow and relative hemoglobin, respectively. With the vacuum set to 125 mmHg, there was a non-significant increase to 230 arbitrary units (*p* = 0.09). After five minutes when the NPWT device was switched off, the arbitrary units decreased to 208 (*p* = 0.09). No change was seen in the relative hemoglobin (*p* = 0.77) (Table 1).

The probes overlying the skin of the contralateral leg showed no overall changes (*p* = 0.82).

**Figure 2:**
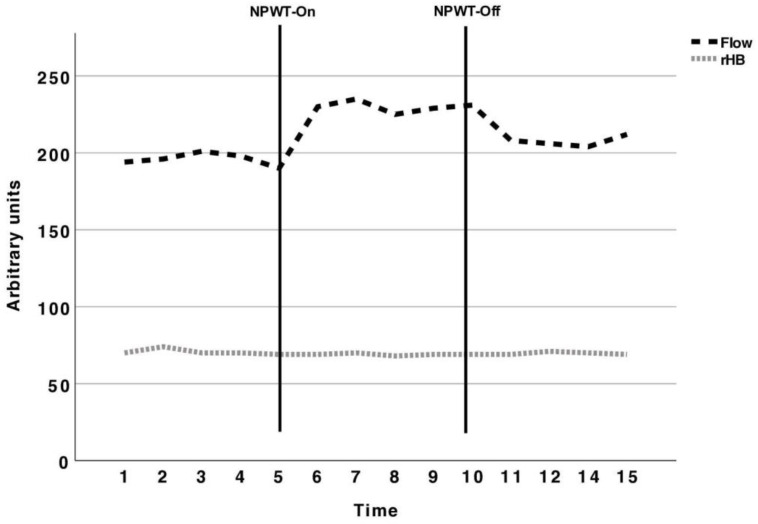
Arbitrary units of the flow and relative hemoglobin (rHB) measured with the O2C from the baseline to NPWT-On and NPWT-Off phases. Continuous vertical bars indicate the borders of each five-minute time period. With NPWT switched on, a non-significant increase in flow and rHB is seen. Similarly, at the ten-minute mark when NPWT was switched off, the values decreased but did not differ significantly from the baseline. The unchanged rHB measured by the O2C most likely depicts the healthy nature of the individuals lacking venous stasis without reflux. The flow changes were non-significant and led to adding the Rad-97^®^ and the in vitro model to our study protocol.

The graphical course of the arbitrary units of flow and relative hemoglobin during the baseline, NPWT-On and NPWT-Off phases is shown in Figure 2.

### 5.2. Perfusion Measurement with the Masimo Rad-97^®^

With the Rad-97^®^, a baseline of 92.91% and 0.17% was measured for the saturation and perfusion index, respectively. No significant change in saturation was noted during the NPWT treatment phase, but the perfusion index increased to 0.32% (*p* = 0.04). After ceasing the NPWT suction, the perfusion index returned back to baseline and did not differ significantly (*p* = 0.72) (Table 2). The contralateral control probe showed no overall changes (*p* = 0.72).

**Figure 3:**
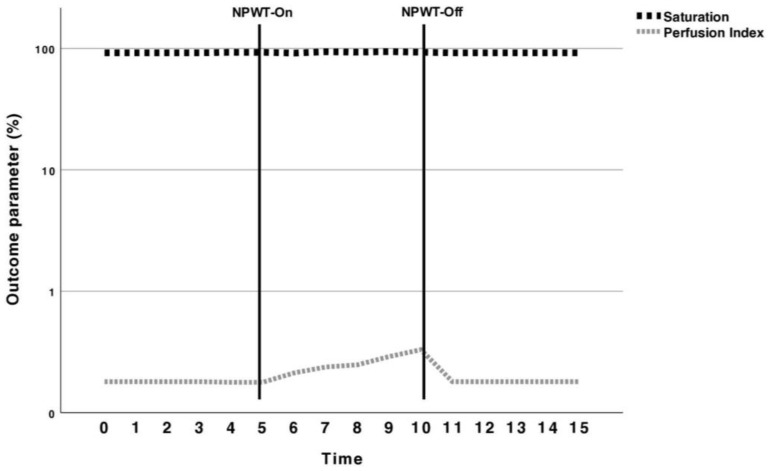
Percentual values on the logarithmic scale of oxygen saturation and perfusion index (PI) measured with the Rad-97^®^ during baseline, NPWT-On and resting phase. Continuous vertical bars indicate the borders of each five-minute time period. The saturation showed no significant changes during the whole course, regardless of the NPWT phase. Contrarily, the perfusion index increased significantly when NPWT was switched on. As the PI depicts the arterial-to-venous perfusion ratio, this might indicate less venous perfusion as a result of compressing the skin with NPWT.

The graphical course of the percentual changes in the saturation and perfusion index during the baseline, NPWT-On and NPWT-Off phases is depicted in Figure 3.

### 5.3. NPWT Device in Arterial and Venous Perfusion

**Figure 4:**
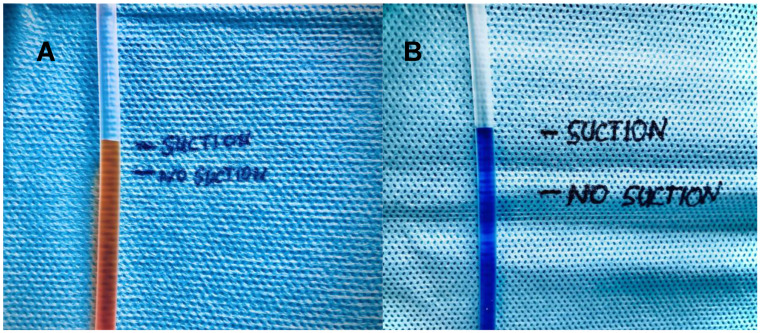
Magnification of water displacement forces on the arterial (**A**) and venous (**B**) system of NPWT at 125mmHG pressure.

Applying the NPWT device over a simple two-vessel model with 125 mmHg suction pressure resulted in a 28 mm and 10 mm increase in the venous and arterial water column, respectively (Figure 4).

## 6. Discussion

For over a decade, the positive effects of negative pressure wound therapy were attributed to an increase in vascular perfusion. Few scientists questioned this effect or doubted the method being used to measure the changes in the microvasculature, that being the laser Doppler [26].

In the presented study, we were able to explain the increased perfusion values of the laser Doppler readings during NPWT using probes equipped with the latest technology. Furthermore, we built an in vitro capillary model in which we were able to determine that the increased flow turned out be an increase in velocity predominantly of the venous system, mediated by the positive pressure applied by NPWT.

Application of positive pressure, via NPWT, to a poorly perfused wound surface has occasionally been concluded to result in a further reduction in perfusion. This, in turn, is in contrast to the positive clinical results that can be observed in many cases with the use of NPWT. From our point of view, the actual processes must be viewed in a more differentiated manner.

Similarly to other recent studies, we found an increase in cutaneous perfusion using the laser Doppler when the vacuum was switched on [19]. Muencho et al. investigated cutaneous blood flow in healthy volunteers during NPTW and used the same measuring device. They found an increase in venous oxygen saturation, blood flow velocity and the relative amount of hemoglobin during the active application of NPWT and the pressure-free interval [27].

Since these results conflict with basic common sense but seem to be safely replicable, their interpretation requires the utmost care. Kairinos et al. previously demonstrated the paradox of negative pressure wound therapy, and showed a positive tissue pressure measured with Codman probes in one-centimeter proximity to an NPWT system [24]. Furthermore, they used technetium and transcutaneous oxygen saturation measurements and found less cutaneous accumulation and oxygen saturation in the extremities of healthy volunteers during NPWT compared to a control [28].

Since these considerations were not taken into account, Kairinos et al. investigated the flaws of laser Doppler measurements and showed increased perfusion when applying the pressure of a fingertip close to a probe. Similarly to kinking a garden hose, the velocity of red blood cells increases and accounts for part of the readings [26].

Our research team was able to confirm these findings and found a surface pressure of 187 mmHg beneath the NPWT system and decreased perfusion in a simple vessel model [1]. However, due to the difficulty of explaining the increased perfusion values of the laser Doppler, we constructed a further two-way explanatory trial. Using the Masimo Rad-97^®^, we were able to differentiate between arterial and venous perfusion and found less venous perfusion when compressing the skin with NPWT. The unchanged rHB measured by the O2C was explained by the healthy nature of the individuals lacking venous stasis without reflux and probe sensitivity. The Masimo Rad-97^®^, however, showed an increased perfusion index, probably due to a change in the arterial-to-venous perfusion ratio. The Rad-97^®^ seems to have a higher sensitivity to and algorithm for small fluctuations in this range.

To more concretely reinforce these findings, we built a static vessel model with NPWT reflecting the different pressure gradients in the arterial and venous systems. The displacing forces on the venous side were greater, confirming the arterial signal enrichment with the Rad-97^®^. The latter has a stronger resistance, which is why the greater effect of NPWT is on the venous system. NPWT thus changes the relationship between the arterial-to-venous perfusion ratio to the disadvantage of the venous system. Among other effects, this obviously leads to less venous pooling and thus to better oxygenation of the tissue.

Such findings may prove beneficial in helping to achieve a better understanding of the underlying mechanism of action with NPWT since similarities can be found to compression therapy in chronic venous insufficiency [29].

Our study has several strengths but also certain limitations since our patient cohort is rather small and there are no comparative studies regarding our vessel model. Unfortunately, the vessel characteristics of veins and stiffer arteries were not respected by our model, adding a potential bias. However, vessels within the terminal vascular bed may easily be compressed by NPWT, regardless of their wall stiffness except for cases with severe atherosclerosis. Furthermore, the algorithm of the Rad-97^®^ to differentiate the PI is rather difficult to understand and rarely investigated in the context of NPWT. Additionally, the probes were placed beneath the foam, adding another variable in terms of local pressure and counter-bearing surface. However, the endorsement of the different measuring systems with the in vitro model shows the most conclusive results.

In summary and the clinical context, the vacuum of the NPWT system mediates a positive pressure to the wound surface and may result in less venous pooling without interfering with arterial nourishment. Additionally, the positive pressure might add to edema reduction and may be one explanation for the overall good results. As the suction intensity and, therefore, the positive pressure are variable, this knowledge may be used in further research to develop high-sensitive sensors to adapt to patient-specific vascular demands and personalize NPWT therapy.

## 7. Conclusions

In conclusion, we were able to identify a possible explanation for the increased perfusion index of the laser Doppler measurements during NPWT. We found a differential displacement of the venous and arterial blood column, with easier displacement of the venous side.

Our in vitro approach showed that compressive forces on the bottom of two water columns within a manometer with different resistances result in unequal displacement.

Similarly to compression therapy in chronic venous insufficiency, a reduction in venous stasis might add to our understanding of the mechanism of action. Further studies investigating the flow component of the laser Doppler are inevitable to better understand this useful diagnostic tool.

## Figures and Tables

**Table 1:** Mean arbitrary units (AUs) of flow and relative hemoglobin (rHB) as measured by the O2C laser Doppler. The values of the NPWT-On and NPWT-Off phases were compared to the baseline (*p*-value in parenthesis).

	Baseline	NPWT-On Phase (*p*-Value of Comparison to Baseline)	NPWT-Off Phase (*p*-Value of Comparison to Baseline)
Flow	194 AUs	230 AUs (0.09)	208 AUs (0.12)
rHB	70 AUs	69 AUs/(0.77)	69.6 AUs (0.74)

**Table 2:** Mean percentual values of saturation and Perfusion Index (PI) measured with the Rad-97^®^. The values of the NPWT-On and NPWT-Off phases were compared to the baseline (*p*-value in parenthesis).

	Baseline	NPWT-On Phase (*p*-Value of Comparison to Baseline)	NPWT-Off Phase (*p*-Value of Comparison to Baseline)
Saturation	92.91%	94.11% (0.21)	93.11% (0.68)
Perfusion Index	0.17%	0.32% (0.04)	0.17% (0.72)

## Data Availability

Dataset available on request from the authors.
